# The relationships between work-family conflict and life satisfaction and happiness among nurses: a moderated mediation model of gratitude and self-compassion

**DOI:** 10.3389/fpubh.2024.1340074

**Published:** 2024-02-21

**Authors:** Mogeda El Sayed El Keshky, Enas ObaidAllah Sarour

**Affiliations:** ^1^Department of Psychology, Faculty of Arts and Humanities, King Abdulaziz University, Jeddah, Saudi Arabia; ^2^Human Sciences and Design, Family Sciences Dept., King Abdulaziz University, Jeddah, Saudi Arabia

**Keywords:** family-work conflict, gratitude, happiness, nurses, life satisfaction, self-compassion, work-family conflict

## Abstract

**Background:**

Researchers and practitioners are concerned with the impact of work-family conflict on the well-being of workers, including those in the health care sector, and previous research suggested that nurses were experiencing a range of negative outcomes.

**Aim:**

To investigate the potentially mediating role of self-compassion and moderating role of gratitude in the relationships between work-family conflict and both life satisfaction and happiness among Saudi nurses.

**Method:**

A cross-sectional survey was conducted with 368 nurses (men age = 35.18, SD = 6.67, 70.65% of females). Nurses were recruited via social media using convenience sampling techniques. They completed the Satisfaction with Life Scale, the Subjective Happiness Scale, the Gratitude Questionnaire–6, the Self-Compassion Scale, and the Work-Family Conflict Scale, as well as a set of demographic questions. The data were analyzed using PROCESS MACRO model 4 and 8, and the reporting followed STROBE checklist.

**Results:**

As expected, the study found a negative relationship between work-family conflict and both life satisfaction and happiness. These relationships were mediated by self-compassion. For the relationships between work-family conflict and life satisfaction and happiness, and between family-work conflict and happiness, this mediation was moderated by gratitude.

**Conclusion:**

This study built on positive psychology and demonstrated that the positive personality traits of self-compassion and gratitude can engender enhanced life satisfaction and happiness among Saudi nurses.

## 1 Introduction

Researchers and workplace organizations have been concerned with the interference between work and family roles for quite some time. It is challenging for workers to achieve a balance between work and family responsibilities (1), which may produce work-family conflict. It has been well-established by prior research that work-family conflict is a threat to the well-being of workers. Many studies have reported direct and indirect negative relationships of work-family conflict with life satisfaction (2–4) (5–8). Prior research has reported additional negative effects of work-family conflict on workers, including burnout (9), physical health problems (10) (11), and psychological issues (12) (13; 11).

Nurses deal with more and greater challenges than many other workers, such as long periods of shift work, heavy workload, and a great deal of pressure (14–16), which may make them more prone to work-family conflict. In studies among nurses, Burke and Greenglass (17), (18), and (19) all reported that work-family conflict was associated with negative well-being effects. Different negative experiences have been reported in Saudi nurses in previous research. Turnover in Saudi nurses were estimated at a rate of 20%, which is higher than other countries such as England (20). In a region of Saudi Arabia, it was reported that nurses had decreased job satisfaction, and a 40% rate of turnover was reported in the study (21). Another study found that Saudi nurses had moderate levels of quality of life (22). It is therefore important to investigate factors that can mitigate this distress among nurses.

Recent research suggests that positive psychology constructs may protect nurses from this distress. A meta-analysis on positive psychology interventions among nurses concluded that mindfulness interventions reduced anxiety among nurses (23). Among healthcare workers, it was reported that mindfulness positively impacted on job satisfaction (24). Another quasi-experimental study reported that nursing students benefited from an empathy intervention program during internship (25). Further, positive psychology constructs, such as hope, resilience, and optimism were found to protect nurses from distress (26). People with high levels of self-compassion used emotion regulation strategies better (27). Self-compassionate people are open to experiences of pain and distress and approach them with self-kindness (28). Further, self-compassion makes people aware of their negative experiences, and they get to acknowledge that other people have these experiences too (29). This means that when self-compassionate individuals experience work-family conflicts, they deal with the situation by being compassionate to themselves, which enhances resilience and well-being.

According to the Socioecological Theory (30), the intersection between individual and contextual variables exerts an influence on behavioral and prosocial outcomes of people. As such, when work-family conflicts impact on the well-being of people through self-compassion, individual variables such as gratitude may be playing a role as well. Grateful individuals have higher odds of experiencing frequent and intense emotions and positive perspectives of their environment, which helps achieve better well-being (31).

Given the links between work-family conflict and negative individual and organizational outcomes, researchers have been concerned with policies, interventions, and practices that can improve workers’ well-being (7, 32, 33). Many of these interventions and programs target the organizational level (34), and interventions for individuals have received comparatively little attention. If indeed workplace organizations do not invest in programs that improve employees’ individual well-being, it is important for workers themselves to find ways to improve their sense of well-being. Positive psychology asserts that there are personal psychological traits and resources that can buffer individuals from negative or stressful circumstances and promote enhanced well-being, and gratitude and self-compassion occupy a central role in these discussions (29, 35). This notion can be applied to reducing work-family conflict (36).

Although previous research has established a negative relationship between work-family conflict and the well-being of workers (7, 37, 38), potential mediating and moderating mechanisms are not fully understood. The purpose of this study was to construct a moderated mediation model according to Hayes (39) in order to test the mediation role of self-compassion and the moderating role of gratitude in the relationship between work-family conflict and happiness and life satisfaction among nurses in Saudi Arabia.

## 2 Literature review

### 2.1 Work-family conflict, life satisfaction, and happiness

As women increasingly enter the labor market while maintaining family and household responsibilities, the incidence of work-family conflict may increase for female workers (40) and indeed for both male and female workers when partners both work and share responsibility for family care-giving (41). In Saudi Arabia, the Brookings Institution estimated an increase of 20 to 33% of women entering the workplace in 2021 (42). Balancing family life and work life has, therefore, been increasingly challenging in Saudi Arabia. This is especially true for nurses whose jobs entail psychological and physical demands associated with heavy workloads, attention to multiple patients, and the need to display positive emotions regardless of their mood (43).

The notion of work-family conflict originates in role theory (44) and the role strain hypothesis (45). Conflict can exist in both directions, work responsibilities interfering with family life (that is, work-family conflict, or WFC) and family responsibilities interfering with work life (family-work conflict, or FWC) (46). Among nurses, it was revealed that work-family conflict acted as a path through which toxic leadership impacted on distress of the nurses (47). To capture a fuller understanding of the interference between work and family responsibilities, researchers have suggested examining both WFC and FWC (33, 48).

As established earlier, WFC has been shown to be a critical factor impacting life satisfaction. Tang et al. (38) reported its negative impact on workers’ happiness, as well. FWC has also been negatively associated with life satisfaction (37) and happiness (49). Studies have highlighted the negative impacts of work-family conflict on nurses’ well-being in particular: WFC has been positively associated with nurses’ turnover intentions (50), job stress (51), and anxiety and depression (52). A negative relationship was also found among nurses between work-family conflict and both life satisfaction (53–55) and job satisfaction (56). Accordingly, the first hypothesis of this study is as follows:

*Hypothesis 1*: WFC and FWC will be negatively associated with life satisfaction and happiness.

### 2.2 The mediating role of self-compassion

The aforementioned research notwithstanding, there have been studies that found no relationship between work-family conflict and life satisfaction (57, 58), which suggests that there may be other pathways through which WFC impacts life satisfaction. Indeed, Taşdelen-Karçkay and Bakalım (54) found a mediating effect of work-life balance in this relationship. This relationship was also reported to be mediated by emotional exhaustion (60) and coping strategies (61). Others have also reported a mediation role of emotional exhaustion in the relationship between work-family conflict and job satisfaction, a construct that is related to life satisfaction among workers (62). However, there have been no studies that investigated the mediation role of self-compassion.

Self-compassion is a positive personality trait that involves treating oneself with a sense of care and kindness rather than self-criticism, especially in the face of adversity and failures (29, 63). Neff (64) adds that self-compassion includes cultivating a sense of connectedness with others rather than of isolation, and a mindful practice of not overly self-identifying with one’s painful thoughts and feelings (64). Self-compassion has been found to benefit individuals’ well-being (65). Self-compassion is linked to adaptive functioning, especially in times of loss, failure, and stress (66). (67) reported that self-compassion is correlated with positive mental health outcomes, such as less depression and anxiety and greater life satisfaction. Neff and Faso (68) reported a positive relationship between self-compassion and well-being in parents of children with autism. And (69) found a negative relationship between self-compassion and work-family interference.

It has been argued that self-compassion protects people when a negative situation is beyond their control or even when they are responsible for the negative circumstances (66); thus, nurses who are self-compassionate may be less affected by work-family conflict regardless of the situation. In previous research specifically with nurses, it was reported that self-compassion was positively associated with job satisfaction and negatively related to burnout (70). Further, self-compassion was an important predictor of relationship satisfaction and conflict resolution (71) and family functioning (72) among nurses. The study’s second set of hypotheses is as follows:

*Hypothesis 2a:* Self-compassion will negatively mediate the relationship between WFC and life satisfaction.

*Hypothesis 2b:* Self-compassion will negatively mediate the relationship between WFC and happiness.

*Hypothesis 2c:* Self-compassion will negatively mediate the relationship between FWC and life satisfaction.

*Hypothesis 2d:* Self-compassion will negatively mediate the relationship between FWC and happiness.

### 2.3 The moderating role of gratitude

Gratitude is the emotion or state of being thankful. In one sense, it can be defined as the emotion that people feel when they receive something that they perceive as valuable, altruistic, or even costly (73), and more broadly can be defined as an attitude or disposition by which one notices and appreciates positive experiences and achievements (74, 75). The broaden-and-build theory (76) posits that positive emotions, including gratitude, help individuals’ build enduring personal physical, intellectual, and psychological resources. It has been postulated that people with high levels of gratitude experience more frequently and intensely positive emotions and use positive coping mechanisms, which enhance their mental well-being (31, 75, 77). Others have claimed that gratitude leads people to interpret other people’s behaviors in a positive way, perceive other people as supportive and available, which benefits both parties (74, 75, 78). Randolph (79) posited that expressing gratitude is essential for nurses to cope with the challenges of work and to enhance family relationships. Prior research has reported that gratitude increased happiness (80), resilience, job satisfaction, and a healthy work environment among nurses (81). Further, gratitude was claimed as beneficial at workplace. In a sample of 411 employees, Mahipalan and Garg (82) reported that gratitude moderated the relationship between workplace bullying and psychological capital of employees. A study involving 112 female employees in India found that gratitude was linked to family enrichment (83), and to spiritual well-being and distress (84). In a sample of healthcare employees in India, Garg et al. (85) concluded that gratitude moderated the relationship between workplace toxicity and turnover intentions. Gratitude was also related to vitality with a mediation of resilience (86). Gratitude was also reported as a moderator in the relationship between teasing and depression (87). Among nurses, gratitude was reported to mitigate the negative impacts of FWC on work engagement (88). Parallelly, it was claimed in previous research that the relationship between self-compassion and happiness was moderated by gratitude (89). Therefore, it is possible that the mediation effect of self-compassion may be different at different levels of gratitude. Accordingly, the third set of hypotheses for our study was as follows:

*Hypothesis 3a:* Gratitude will moderate the relationship between WFC and self-compassion.

*Hypothesis 3b:* Gratitude will moderate the relationship between WFC and life satisfaction.

*Hypothesis 3c:* Gratitude will moderate the relationship between WFC and happiness.

*Hypothesis 3d:* Gratitude will moderate the relationship between FWC and self-compassion.

*Hypothesis 3e:* Gratitude will moderate the relationship between FWC and life satisfaction.

*Hypothesis 3f:* Gratitude will moderate the relationship between FWC and happiness.

## 3 Method

### 3.1 Sample and procedure

Nurses were recruited via social media using convenience sampling techniques. The design of this study is cross-sectional. The questionnaire was sent to respondents via email, Facebook, WhatsApp, and Twitter. Nurses were approached through their hospitals whose managers provided their emails; nurses were then asked to send the link of the questionnaire to other nurses they knew via different media platforms. The data were collected from December 10, 2022 to January 28, 2023 in the Kingdom of Saudi Arabia. A total number of 425 participants returned the completed survey, but only 368 surveys without missing data were used in the study. The mean age of the surveyed nurses was 35.18 with a standard deviation of 6.67. 70.65% were female, 28.2% were single, 65% were married, 5.7% divorced, and 1.1% widowed. About 34.2% had a diploma degree, 8.7% had a diploma after university degree, 52.7% had a university degree, 3.8% had a master’s degree, and 0.6% possessed a doctorate. Around 83.4% were working in the government sector, 3.6% in a semi-government sector, and 13% were working in the private sector. Around 90.2% were full-time workers, 2.7% part-time, and 7.1% were working irregular hours. About 5.2% had less than 1 year of experience, 5.7% had 1 to 3 years of experience, 16.3% 3 to 6 years, 16% between 6 and 9 years, 21.2% between 9 and 12 years, 13% between 12 and 15 years, and 22.6% had more than 15 years of experience. Around 8.7% had monthly income of less than 5,000 RS (Saudi Riyal), 31.2% had income between 5,000 and 9,000 SR, 31.5% between 9,000 and 13,000 SR, 17.4% between 13,000 and 17,000 SR, 5.4% between 17,000 and 21,000, 4% between 21,000 and 25,000, and 1.6% had monthly income greater than 25,000 SR. Finally, 62% were Saudi citizens, 22.8% were residents from non-Arab countries, and 15.2% were residents from other Arab countries.

### 3.2 Measures

Study participants completed questionnaires which included a set of demographic questions as well as the Satisfaction with Life Scale (90), the Subjective Happiness Scale (91), the Gratitude Questionnaire–6 (GQ-6) (74), the Self-Compassion Scale (92), and the Work-Family Conflict Scale (93).

#### 3.2.1 The satisfaction with life scale

This is a five-item measure for self-evaluations of overall satisfaction with one’s life. This scale is measured on a 7-point Likert scale, ranging from 1 (strongly disagree) to 7 (strongly agree) for each item (90). Thus, total scores range from 5 to 35. In this study, the scale exhibited a good internal consistency reliability (*α* = 0.85).

#### 3.2.2 The subjective happiness scale

This measure consisted of four-item measure scored on a 7-point Likert scale. Each item presents the respondent with a sentence fragment and two polar characterizations that complete the sentence. Respondents select a number from 1 to 7 to indicate the extent to which the characterizations describe themselves.

Total scores for the Subjective Happiness Scale range from 4 to 28 (91). In this study, the scale had adequate internal consistency reliability (*α* = 0.65).

#### 3.2.3 The gratitude questionnaire–6

This is a six-item scale that measures respondents’ self-evaluations of gratitude. Each item of the scale is scored on a 7-point Likert scale, ranging from 1 (strongly disagree) to 7 (strongly agree). Thus, total scores range from 6 to 42. Items 3and 6 are reversely scored (74). This scale exhibited good internal consistency reliability (*α* = 0.73).

#### 3.2.4 The self-compassion scale

This scale consisted of 12-item instrument designed to measure how compassionate people are with themselves and is score on a 5-point Likert scale, ranging from 1 (almost never) to 5 (almost always) for each item. Total scores range from 12 to 60 (92). This scale exhibited an acceptable internal consistency reliability (*α* = 0.74).

#### 3.2.5 The work-family conflict scale

This is a short instrument comprised of two related subscales with five items each: the work-to-family conflict subscale (WFC) and the family-to-work conflict subscale (FWC). Each item is rated on a 7-point Likert scale, ranging between 1 (very strongly disagree) and 7 (very strongly agree). The total scores for each subscale range between 5 and 35 (93). The scale exhibited adequate internal consistency reliability with (*α* = 0.82 for WFC and *α* = 0.79 for FWC).

### 3.3 Statistical analysis

All the data analyses were conducted using the RStudio software (94). For much of the analysis, an add-on software package called Process, developed by Hayes (39), was used. Descriptive statistics and Pearson correlations between the study variables were gathered first. Secondly, a mediation analysis was conducted using Model 4 of the Process software, and a moderated mediation analysis was performed using Process Model 8. We used 95% confidence intervals with 10,000 bootstrap samples. 95% confidence intervals that do not contain zero indicate statistically significant conditional indirect effects. The Process software also allows centering of variables that are part of the product terms, to set conditional effects for different levels of the moderator variables (mean-1SD, mean, and mean + 1SD), and to bootstrap results against violations of normality and homoscedasticity assumptions. It has been established by previous studies that age and gender were significantly associated with life satisfaction and happiness (95–97); therefore, we included these variables as covariates.

### 3.4 Ethics

All procedures followed were in accordance with the ethical standards of the responsible committees on human experimentation (institutional and national) and with the Helsinki Declaration of 1975, as revised in 2000. Approval for conducting this study was obtained from the ethics committee of Institutional Review Board of King Abdulaziz University, Jeddah in Saudi Arabia (No 343-253-1443). Informed consent was obtained from all participants.

## 4 Results

### 4.1 Descriptive statistics and Pearson correlations

The results of the descriptive statistics and the Pearson correlations are summarized in Table 1. The mean scores of participants on the scales were as follows: 24.15 for life satisfaction (SD = 6.48, range = 5–35), 31.7 for gratitude (SD = 5.75, range = 5–42), 20.09 for WFC (SD = 5.62, range = 5–35), 14.71 for FWC (SD = 7.14, range = 5–35), 41.73 for self-compassion (SD = 7.02, range = 12–60), and 19.31 for happiness (SD = 4.10, range = 4–28). As expected, life satisfaction was positively correlated with gratitude, self-compassion, and happiness, and negatively correlated with WFC and FWC. Happiness was positively correlated with life satisfaction, gratitude and self-compassion, and negatively correlated with WFC, and slightly with FWC. Finally, gratitude and self-compassion were positively correlated.

**Table 1:** Descriptive statistics and Pearson correlations between study variables.

	Mean	SD	1	2	3	4	5	6
1. Life satisfaction	24.15	6.48	1					
2. Gratitude	31.7	5.75	0.52***	1				
3. WFC	20.09	5.62	−0.24***	−0.22***	1			
4. FWC	14.71	7.14	−0.26***	−0.22***	0.60***	1		
5. Self-compassion	41.73	7.02	0.35***	0.43***	−0.30***	−0.31***	1	
6. Happiness	19.31	4.10	0.33***	0.50***	−0.12**	−0.09*	0.51***	1

WFC, work-to-family conflict; FWC, family-to-work conflict; *p* < 0.05; **p < 0.01; ***p < 0.001. Numbered columns represent the traits shown with the corresponding row number.

### 4.2 Testing for the mediation model

To answer Hypothesis 1, that is, *WFC and FWC will be negatively associated with life satisfaction and happiness*, we used the mediation analysis module of the Process software package with gender and age as covariates. The results show that WFC was negatively associated with life satisfaction (*β* = −0.15, *p* < 0.05, 95% CI [−0.21 to −0.09]) and with happiness (*β* = −0.07, *p* < 0.05, 95% CI [−0.12 to −0.03]) in the absence of a mediator. FWC was similarly negatively related to life satisfaction (*β* = −0.16, *p* < 0.01, 95% CI [−0.22 to −0.11]) and happiness (*β* = −0.03, *p* < 0.05, 95% CI [−0.11 to −0.03]) in the absence of a mediator. Therefore, Hypothesis 1 was supported.

When the mediator was included, WFC was negatively associated with life satisfaction (*β* = −0.15, *p* < 0.01, 95% CI [−0.29 to −0.06]), but not with happiness. Similarly, FWC was negatively associated with life satisfaction (*β* = −0.17, *p* < 0.01, 95% CI [−0.25 to −0.07]) but not with happiness. Self-compassion was related to life satisfaction (*β* = 0.29, *p* < 0.001, 95% CI [0.17 to 0.35]), happiness (*β* = 0.29, *p* < 0.001, 95% CI [0.23 to 0.34]), WFC (*β* = −0.30, *p* < 0.001, 95% CI [−0.50 to −0.26]), and FWC (*β* = −0.31, *p* < 0.001, 95% CI [−0.40 to −0.21]). As shown in Table 2, these relationships were established in the presence of covariates.

**Table 2:** Mediation effects of work-family conflict on life satisfaction and happiness.

Outcome variable	Independent variable	*β*	SE	*t*	*p*	LLCI	ULCI
Life satisfaction	Constant	24.41	2.02	12.04	<0.001	12.32	28.43
	WFC	−0.15	0.07	−2.17	<0.05	−0.09	−0.21
	FWC	−0.16	0.05	−2.94	<0.01	−0.11	−0.22
	Age	0.15	0.04	3.17	<0.01	0.08	0.19
	Gender	−0.08	0.71	−0.58	0.556	−0.11	0.02
Happiness	Constant	17.22	1.31	13.11	<0.001	13.32	23.45
	WFC	−0.07	0.04	−1.61	<0.05	−0.12	−0.03
	FWC	−0.03	0.03	−0.39	<0.05	−0.11	−0.01
	Age	0.11	0.01	3.76	<0.001	0.05	0.16
	Gender	−0.21	0.46	−2.82	<0.01	−0.34	−0.11
Life satisfaction	Constant	13.27	2.87	4.61	<0.001	7.62	18.62
	WFC	−0.15	0.05	−3.11	<0.01	−0.29	−0.06
	Self-compassion	0.29	0.04	5.59	<0.001	0.17	0.36
	Age	0.10	0.04	2.03	<0.05	0.003	0.19
	Gender	−0.01	0.70	−0.33	0.740	−1.61	1.15
Happiness	Constant	4.18	1.68	2.48	<0.001	0.86	7.49
	WFC	0.03	0.03	0.73	0.465	−0.04	0.09
	Self-compassion	0.29	0.02	10.36	<0.001	0.23	0.34
	Age	0.05	0.02	1.92	0.05	−0.001	0.11
	Gender	0.07	0.41	1.68	0.09	−0.11	1.50
Self-compassion	Constant	40.15	2.31	17.31	<0.001	35.5	44.7
	WFC	−0.30	0.06	−6.30	<0.001	−0.50	−0.26
	Age	0.21	0.05	4.27	<0.001	0.11	0.32
	Gender	0.13	0.75	2.75	<0.01	0.09	0.34
Life satisfaction	Constant	12.30	2.63	4.66	<0.001	7.11	17.49
	FWC	−0.17	0.04	−3.49	<0.001	−0.25	−0.07
	Self-compassion	0.28	0.04	5.45	<0.001	0.16	0.35
	Age	0.09	0.04	1.96	0.05	−0.0002	0.19
	Gender	−0.006	0.70	−0.13	0.894	−1.47	1.28
Happiness	Constant	3.82	1.54	2.46	<0.01	0.77	6.86
	FWC	0.09	0.02	1.54	0.124	−0.01	0.11
	Self-compassion	0.29	0.02	10.62	<0.001	0.24	0.35
	Age	0.08	0.02	1.90	0.05	−0.001	0.10
	Gender	0.07	0.41	1.59	0.110	−0.15	1.46
Self-compassion	Constant	37.23	2.11	17.5	<0.001	33.07	41.39
	FWC	−0.31	0.04	−6.46	<0.001	−0.40	−0.21
	Age	0.19	0.05	4.05	<0.001	0.10	0.31
	Gender	0.15	0.75	3.10	<0.01	0.011	0.021

*β* values are standardized coefficients; LLCI, low limit of confidence interval; ULCI, upper limit of confidence interval.

To test the mediation model, we used the bootstrap method and the results are summarized in Table 3. The indirect effect of self-compassion was −0.10 (95% CI = −0.13 to −0.04) on the relationship between WFC and life satisfaction, −0.08 (95% CI = −0.14 to −0.04) on the relationship between FWC and life satisfaction, −0.15 (95% CI = −0.21 to −0.09) on the relationship between WFC and happiness, and − 0.16 (95% CI = −0.22 to −0.10) on the relationship between FWC and happiness. None of these confidence intervals contained the value zero, which indicates that the indirect effects were statistically significant. Therefore, our hypotheses that *self-compassion will mediate these relationships* (Hypotheses 2a, 2b, 2c, and 2d) were supported.

**Table 3:** Bootstrapping indirect effect and 95% confidence interval (CI) for the mediation model.

Indirect path	Standardized estimated effect	BootSE	95% CI
WFC= > self-compassion= > life satisfaction	−0.10	0.02	[−0.13, −0.04]
FWC= > self-compassion= > life satisfaction	−0.08	0.02	[−0.14, −0.04]
WFC= > self-compassion= > happiness	−0.15	0.02	[−0.21, −0.09]
FWC= > self-compassion= > happiness	−0.16	0.02	[−0.22, −0.10]

BootSE, bootstrapping standard error; CI, confidence intervals.Confidence intervals that do not contain 0 are significant.

### 4.3 Testing for the moderated mediation model

To check the *gratitude as moderator* hypotheses (3a through 3f), we used the moderated mediation analysis module of the Process software package with gender and age as covariates. WFC was negatively associated with self-compassion (*β* = −0.28, *p* < 0.001), and the interaction with gratitude was statistically significant (*β* = −0.03, *p* < 0.001). Thus, Hypothesis 3a was supported. WFC was also negatively associated with life satisfaction (*β* = −0.13, *p* < 0.05), but the interaction with gratitude was not statistically significant. Thus, Hypothesis 3b was not supported. On the other hand, WFC was not related to happiness and the interaction with gratitude was also not statistically significant. Therefore, Hypothesis 3c was also not supported. FWC was negatively related to self-compassion (*β* = −0.21, *p* < 0.001) and the interaction with gratitude was not statistically significant. Therefore, Hypothesis 3d was not supported. FWC was negatively related to life satisfaction (*β* = −0.12, *p* < 0.01) and the interaction with gratitude was not statistically significant. Therefore, Hypothesis 3e was also not supported. Lastly, FWC was negatively associated with happiness (*β* = −0.06, *p* < 0.01) and the interaction with gratitude was statistically significant as well (*β* = −0.009, *p* < 0.01). Thus, Hypothesis 3 was supported (Table 4).

**Table 4:** Moderated mediation effect of work-family conflict on life satisfaction and happiness.

	Independent variable	*β*	SE	*t*	*p*	LLCI	ULCI
**Mediator variable model**							
Self-compassion	Constant	33.88	1.82	18.56	<0.001	30.29	37.47
	WFC	−0.28	0.05	−5.03	<0.001	−0.39	−0.17
	Gratitude	0.42	0.05	7.41	<0.001	0.40	0.53
	WFC x gratitude	−0.03	0.009	−4.09	<0.001	−0.01	−0.005
	Age	0.19	0.04	4.09	<0.001	0.10	0.28
	Gender	0.08	0.69	1.55	0.121	−0.01	0.10
**Dependent variable model**							
Life satisfaction	Constant	15.47	2.31	6.69	<0.001	10.92	20.01
	WFC	−0.13	0.05	−2.56	<0.05	−0.23	−0.03
	Self-compassion	0.10	0.04	2.21	<0.05	0.01	0.19
	Gratitude	0.54	0.05	9.78	<0.001	0.43	0.64
	WFC x gratitude	0.01	0.003	1.33	0.181	−0.005	0.02
	Age	0.14	0.04	3.38	<0.001	0.06	0.23
	Gender	−0.06	0.63	−1.93	0.05	−0.10	0.01
Happiness	Constant	7.80	1.41	5.50	<0.001	5.01	10.59
	WFC	0.04	0.03	1.44	0.148	−0.01	0.11
	Self-compassion	0.20	0.02	7.11	<0.001	0.15	0.26
	Gratitude	0.25	0.03	7.52	<0.001	0.18	0.32
	WFC x gratitude	0.00	0.005	0.004	0.996	−0.01	0.01
	Age	0.07	0.02	2.81	<0.01	0.02	0.12
	Gender	0.24	0.38	0.62	0.531	−0.52	0.92
**Mediator variable**							
Self-compassion	Constant	33.1	1.85	17.8	<0.001	29.48	36.78
	FWC	−0.21	0.04	−4.83	<0.001	−0.30	−0.12
	Gratitude	0.44	0.05	7.69	<0.001	0.33	0.55
	FWC x gratitude	−0.01	0.008	−1.48	0.137	−0.02	0.004
	Age	0.21	0.04	4.56	<0.001	0.12	0.31
	Gender	0.09	0.71	1.67	0.09	−0.06	0.12
**Dependent variable model**							
Life satisfaction	Constant	16.32	2.22	7.22	<0.001	11.8	20.7
	FWC	−0.12	0.04	−2.97	<0.01	−0.20	−0.04
	Self-compassion	0.09	0.04	1.96	0.05	−0.0001	0.18
	Gratitude	0.53	0.05	9.67	<0.001	0.42	0.64
	FWC*gratitude	0.007	0.007	0.92	0.353	−0.0079	0.02
	Age	0.13	0.04	3.15	<0.01	0.05	0.22
	Gender	−0.09	0.63	−1.71	0.08	−0.15	−0.01
Happiness	Constant	7.56	1.37	5.51	<0.001	4.86	10.28
	FWC	−0.06	0.02	−2.63	<0.01	−0.11	−0.01
	Self-compassion	0.21	0.02	7.41	<0.001	0.15	0.26
	Gratitude	0.25	0.03	7.45	<0.001	0.18	0.41
	FWC*gratitude	−0.009	0.004	−1.26	<0.05	−0.019	−0.0009
	Age	0.07	0.02	2.99	<0.01	0.02	0.12
	Gender	0.21	0.38	0.56	0.569	−0.53	−0.97

SE, standard errors; LLCI, low limit of confidence interval; ULCI, upper limit of confidence interval.

The indirect effects of self-compassion at different levels of gratitude are summarized in Table 5. The indirect effect of self-compassion on the relationship between WFC and life satisfaction was not statistically significant for participants with low levels of gratitude (*β*_ind_ = −0.009, 95% CI = −0.002 to 0.0007), but was statistically significant for participants with medium levels of gratitude (*β*_ind_ = −0.02, 95% CI = −0.06 to −0.001) and with higher levels of gratitude (*β*_ind_ = −0.05, 95% CI = −0.011 to −0.003), which indicates that the indirect effect of self-compassion on the relationship between WFC and life satisfaction was stronger for those with higher levels of gratitude. Similarly, the indirect effect of self-compassion on the relationship between WFC and happiness was not statistically significant for participants with low levels of gratitude (*β*_ind_ = −0.01, 95% CI = −0.04 to 0.01), but was statistically significant for participants with medium levels of gratitude (*β*_ind_ = −0.05, 95% CI = −0.08 to −0.03) and higher levels of gratitude (*β*_ind_ = −0.10, 95% CI = −0.015 to −0.06). Thus, the indirect effect of self-compassion on the relationship between WFC and happiness was also stronger for those with higher levels of gratitude.

**Table 5:** Conditional indirect effects of work-family conflict on life satisfaction and happiness for different levels of gratitude.

	Classification	Indirect effects	BootSE	95% CI
WFC= > self-compassion= > life satisfaction	M_gratitude_-1 SD	−0.006	0.009	[−0.002, 0.0007]
	M_gratitude_	−0.02	0.01	[−0.06, −0.001]
	M_gratitude_+1 SD	−0.05	0.02	[−0.011, −0.003]
FWC= > self-compassion= > life satisfaction	M_gratitude_-1 SD	−0.01	0.01	[−0.03, 0.0008]
	M_gratitude_	−0.02	0.01	[−0.04, 0.0006]
	M_gratitude_+1 SD	−0.02	0.01	[−0.06, 0.0007]
WFC= > self-compassion= > happiness	M_gratitude_-1 SD	−0.01	0.01	[−0.04, 0.01]
	M_gratitude_	−0.05	0.01	[−0.08, −0.03]
	M_gratitude_+1 SD	−0.10	0.02	[−0.15, −0.06]
FWC= > self-compassion= > happiness	M_gratitude_-1 SD	−0.03	0.01	[−0.06, −0.003]
	M_gratitude_	−0.04	0.01	[−0.07, −0.02]
	M_gratitude_+1 SD	−0.06	0.01	[−0.09, −0.03]

BootSE, bootstrapping standard error; CI, confidence intervals.Confidence intervals that do not contain 0 are significant.

**Figure 1:**
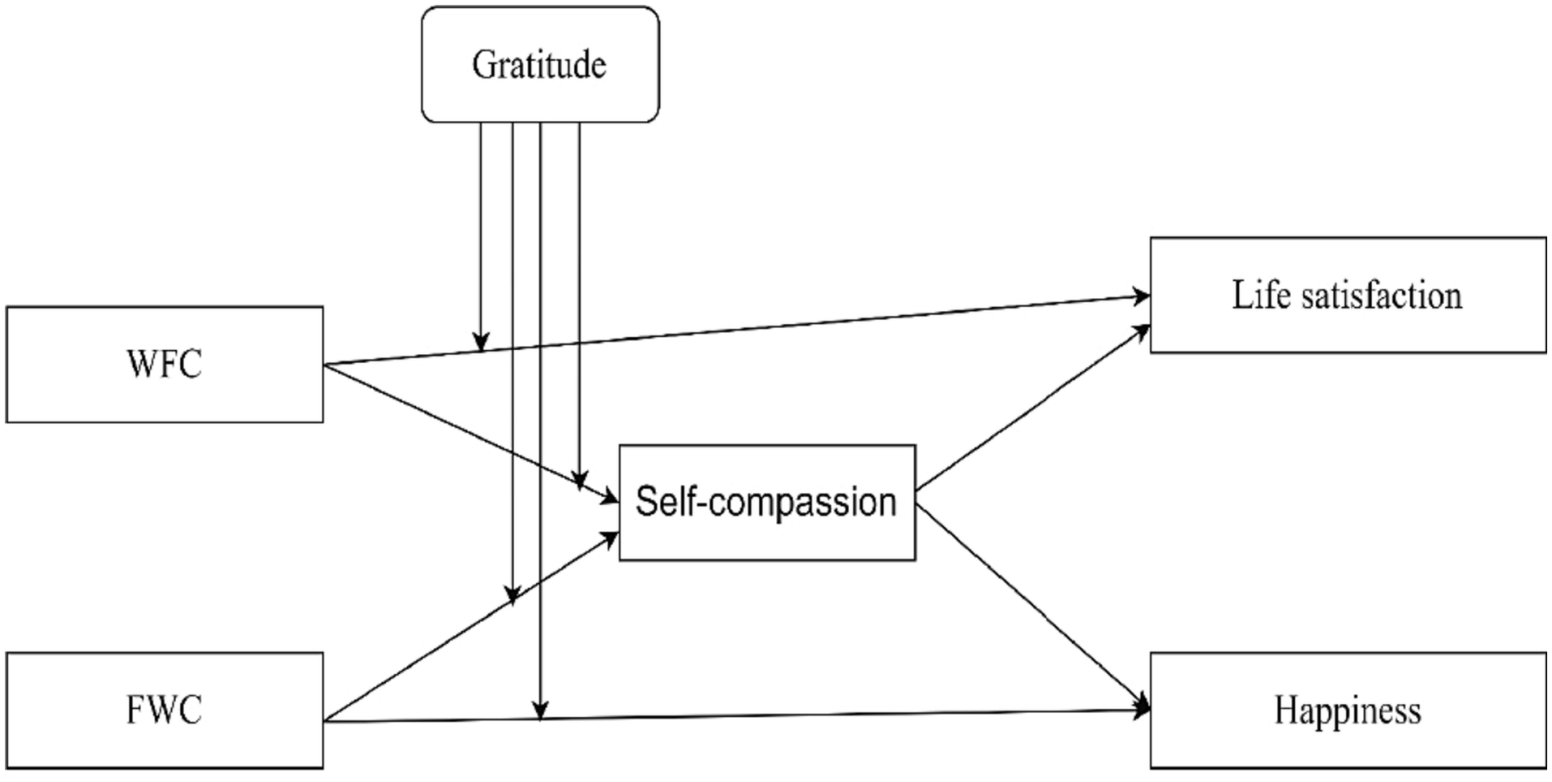
Conceptual Research Model.

**Figure 2:**
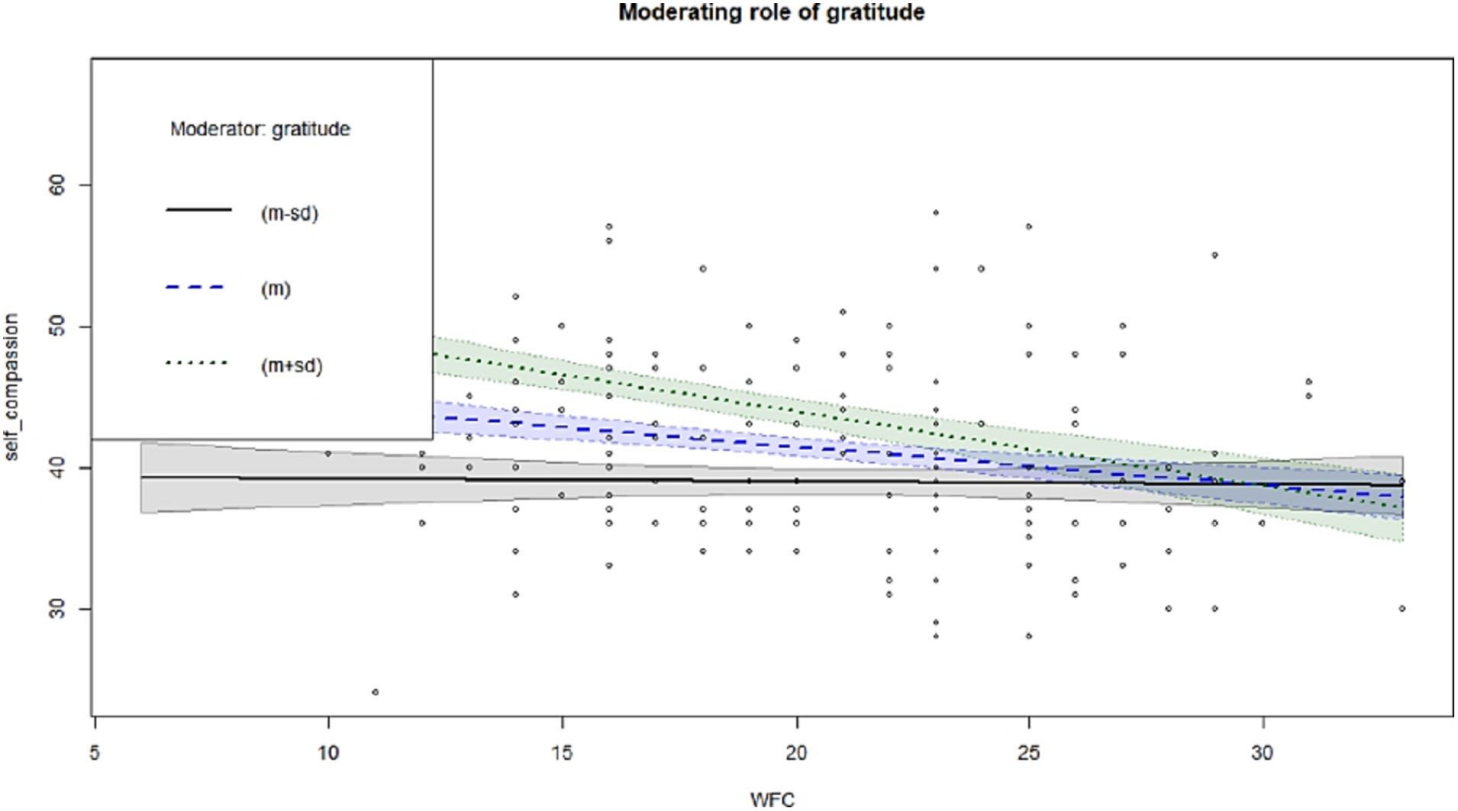
Relationship between FWC and self-compassion for 3 gratitude conditions.

The indirect effect of self-compassion on the relationship between FWC and life satisfaction was not statistically significant for participants with low levels of gratitude (*β*_ind_ = −0.01, 95% CI = −0.03 to 0.0008), medium levels of gratitude (*β*_ind_ = −0.02, 95% CI = −0.04 to 0.0006) or higher levels of gratitude (*β*_ind_ = −0.02, 95% CI = −0.06 to 0.0007). The indirect effect of self-compassion on the relationship between FWC and happiness was statistically significant for participants with low levels of gratitude (*β*_ind_ = −0.03, 95% CI = −0.06 to −0.003), medium levels of gratitude (*β*_ind_ = −0.04, 95% CI = −0.07 to −0.02) and higher levels of gratitude (*β*_ind_ = −0.06, 95% CI = −0.09 to −0.03). Thus, the indirect effect of self-compassion on the relationship between WFC and life satisfaction was beneficial for all levels of gratitude. These conditions are plotted in Figures 1, 2.

## 5 Discussion

This study demonstrated moderating and mediating mechanisms impacting the association between work-family conflict and happiness and life satisfaction among a sample of Saudi nurses. The main findings indicated a statistically significant negative relationship of WFC and FWC with both life satisfaction and happiness. The results also showed that self-compassion negatively mediated the relationship between WFC and life satisfaction, between WFC and happiness, between FWC and life satisfaction, and between FWC and happiness. The results also indicated that gratitude moderated the mediation of self-compassion in the relationship between WFC and life satisfaction, between WFC and happiness, and between FWC and happiness. These results yield theoretical and practical implications for the well-being of workers in general and nurses in particular.

The negative relationships found between work-family conflict and both life satisfaction and happiness corroborate prior research enumerated earlier in this paper. However, some studies reported no statistically significant association between work-family conflict and life satisfaction (57, 58). This inconsistency in findings may suggest cultural differences or other mechanisms underlying or impacting these relationships. This study found that these relationships were negatively mediated by self-compassion, such that the impacts of WFC and FWC on life satisfaction and happiness were mitigated through self-compassion. (59) reported that self-compassion diminishes the emotional exhaustion dimension of work burnout, and a longitudinal study by Schabram and Heng (98) found that self-compassion was a remedy for work-related emotional exhaustion. Others have reported that self-compassion enables people to enhance their emotional resilience (99), which enhances well-being. Self-compassion incorporates evaluations of unpleasant situations with a sense of tolerance and understanding, which may explain why self-compassionate individuals are able to find happiness and satisfaction in their lives (100). Accordingly, nurses who experience WFC and FWC may nonetheless be happier and satisfied with their lives if they are self-compassionate.

We found that this mediation of self-compassion varied with differing levels of gratitude. The results suggest that self-compassion strongly mitigated the negative impacts of work-family conflict on well-being for nurses with high levels of gratitude. The explanation may be the fact that people who are grateful take better care of themselves, are more compassionate and kind, are more resilient in times of adversity, and have healthier sleep (79). According to the broaden-and-build theory (101), when individuals experience positive emotions such as gratitude, they build cognitive and emotional resources needed to deal with any circumstance. Fredrickson and Joiner (102) claimed these positive resources help individuals to develop and grow, and lead to effective responses to new circumstances, including work and family situations and conditions. The efforts of this study speak, therefore, to the call of Nicklin et al. (36) to consider gratitude among the positive resources that can facilitate healthy work-family balance.

This study joins previous theories, the role theory (44) and the role strain theory (45) and postulates that conflicting roles at work and at family create strain for employees, and yet joins again the broaden-and-build theory (76) and socioecological theory (30) to posit that gratitude and self-compassion create lasting physical and psychological resources that can mitigate the strain and facilitate employees’ well-being.

### 5.1 Implications of the study

This study has theoretical and practical implications. For theoretical implications, this study contributed to the literature by establishing evidence of the role of self-compassion and gratitude in protecting nurses from work-family conflicts among a Saudi sample. These findings have practical implications for organizations in general and hospitals in particular. For hospital HR, it is important to plan programs to enhance self-compassion and gratitude of the nurses. These positive psychology interventions have proved their effectiveness, and it is crucial that Saudi hospitals plan effective interventions to promote nurses’ well-being, which would increase their performance. Gratitude and self-compassion are trainable and can therefore be practiced and enhanced, and they present affordable targets for interventions to enhance the well-being of workers in general and nurses in particular. Therefore, managers should consider bringing into the workplace positive psychological interventions focusing on growing gratitude and self-compassion. Further, workers themselves should learn about these positive interventions and attempt to integrate them into their psychological, work, and family lives.

### 5.2 Limitations

Despite the contributions of this study, there are limitations that should be mentioned. First, the design was cross-sectional. Longitudinal designs are recommended for future research. Second, the study used convenience sampling and may have yielded sampling and selection bias, which hampers generalizability of findings; random sampling is recommended for future research. Third, the proportion of females was relatively high in the sample and it is advised that a more proportionate sample be tested. Fourth, the Subjective Happiness Scale has a relatively low Cronbach’s alpha and results should be interpreted bearing this in mind. Fifth, the data collection was made via online platforms, we cannot know in which conditions the respondents were when the completed the questionnaire. Further, the recruiting process via social media is beyond any control of who has participated and what the respondents are representing. Future research should use also other means of data collection to facilitate data cross-validation. Sixth, this study relied on subjective data, future research should include also objective measures.

## 6 Conclusion

Work-family conflict remains an important concern for the well-being of workers and for the performance organizations that employ them. Previous research has established different pathways through which these relationships operate. This study built on positive psychology and investigated the underlying mediating role of self-compassion and moderating role of gratitude in the relationships between work-family conflict and life satisfaction and happiness among Saudi nurses. The results showed a mediation role of self-compassion on the impact of work-family conflict on the well-being of nurses. Moreover, this mediation was moderated by gratitude. Gratitude and self-compassion are positive constructs that are able to bring positive benefits to the lives of nurses and workers in general. These findings suggest that interventions and programs targeted at enhancing the well-being of nurses should include these positive psychology constructs, self-compassion and gratitude. Nurses are also advised to seek to grow self-compassion and gratitude within themselves, since they are trainable and teachable and can be self-administered. The current healthcare system seems to be built on the exploitation of HCPs, following the premise that they will find their own ways to optimize their resilience and endurance. However, without changes in the organizational structure, this demand for self-optimization is self-limiting and will easily result in “organized” burnout.

## Data availability statement

The raw data supporting the conclusions of this article will be made available by the authors, without undue reservation.

## Ethics statement

Approval for conducting this study was obtained from the ethics committee of Institutional Review Board of King Abdulaziz University, Jeddah in Saudi Arabia (No 343-253-1443). Informed consent was obtained from all participants.

## Author contributions

ME: Conceptualization, Data curation, Formal analysis, Investigation, Methodology, Project administration, Resources, Software, Supervision, Validation, Visualization, Writing – original draft, Writing – review & editing. ES: Data curation, Funding acquisition, Investigation, Writing – review & editing.
